# Micro-Economic Impact of Congenital Heart Surgery: Results of a Prospective Study from a Limited-Resource Setting

**DOI:** 10.1371/journal.pone.0131348

**Published:** 2015-06-25

**Authors:** Manu Raj, Mary Paul, Abish Sudhakar, Anu Alphonse Varghese, Aareesh Chittulliparamb Haridas, Conrad Kabali, Raman Krishna Kumar

**Affiliations:** 1 Department of Pediatric Cardiology, Amrita Institute of Medical Sciences and Research Centre, Kochi, Kerala, India; 2 Consultant Epidemiologist, Dalla Lana School of Public Health, University of Toronto, Toronto, ON Canada; University of Washington, UNITED STATES

## Abstract

**Introduction:**

The microeconomic impact of surgery for congenital heart disease is unexplored, particularly in resource limited environments. We sought to understand the direct and indirect costs related to congenital heart surgery and its impact on Indian households from a family perspective.

**Methods:**

Baseline and first follow-up data of 644 consecutive children admitted for surgery for congenital heart disease (March 2013 – July 2014) in a tertiary referral hospital in Central Kerala, South India was collected prospectivelyfrom parents through questionnaires using a semi-structured interview schedule.

**Results:**

The median age was 8.2 months (IQR: 3.0– 36.0 months). Most families belonged to upper middle (43.0%) and lower middle (35.7%) socioeconomic class. Only 3.9% of families had some form of health insurance. The median expense for the admission and surgery was INR 201898 (IQR: 163287–266139) [I$ 11989 (IQR: 9696–15804)], which was 0.93 (IQR: 0.52–1.49) times the annual family income of affected patients. Median loss of man-days was 35 (IQR: 24–50) and job-days was 15 (IQR: 11–24). Surgical risk category and hospital stay duration significantly predicted higher costs. One in two families reported overwhelming to high financial stress during admission period for surgery. Approximately half of the families borrowed money during the follow up period after surgery.

**Conclusion:**

Surgery for congenital heart disease results in significant financial burden for majority of families studied. Efforts should be directed at further reductions in treatment costs without compromising the quality of care together with generating financial support for affected families.

## Introduction

Congenital heart disease (CHD) accounts for nearly one-third of all major congenital anomalies with a prevalence rate of 9.3 per 1000 live births in Asia [1]. Children with congenital heart disease often require surgical or interventional treatments and continued medical care throughout their life. Even though medical and surgical advancements have simplified the management of congenital heart disease, it has also become more expensive. The hospital costs related to congenital cardiovascular anomalies is reported to be more than half of all hospital costs for birth defects [2].

The cost as experienced by parents of children with CHD is often life changing and uncertain [3]. Complexity of congenital heart surgery and length of hospital stay (LOS) are known to increase the hospital costs related to congenital heart disease surgeries [4, 5].

In India, delivering quality care at a cost that is affordable to the majority of affected families with CHD is still a challenge [6]. The overall economic burden experienced by the parents who have a child undergoing congenital heart surgery is unknown in most situations and has not been explored in resource limited environments.

Economic analysis of impact of ill health complements clinical or epidemiological approaches to disease burden assessment and can help address important policy questions concerning the consequences of disease [7]. In this era of rising health care costs, understanding the health economics of congenital heart surgery is important to develop initiatives aimed at cost reduction and to introduce support schemes for the beneficiaries.

The primary objective of the study was to understand the financial aspects related to cost of congenital heart surgery from the perspective of individual families. The secondary objectives were to identify the predictors of higher cost and to create a prediction model for total hospital costs.

## Methods

### Study Setting and Design

The study was done in a tertiary referral hospital in Central Kerala, India. The Pediatric Cardiology and Pediatric Cardiac Surgery Departments in the hospital offers pediatric cardiac services and perform approximately 700 surgeries for children with congenital heart disease every year. Children and adolescents belonging to lower socio economic levels who come for treatment of cardiac illness including surgery are eligible for subsidized or free care in the hospital.

Inclusion criteria: All children in the age group 0–18 years who were admitted in the hospital for congenital heart disease surgery and their parents were included in the study. Exclusion criteria: Children admitted for catheter based interventions were excluded. Foreign nationals and children from states other than those living in Kerala and the neighbouring state of Tamil Nadu were also excluded because of anticipated difficulties with follow-up. Data of those patients who expired during the postoperative hospital stay (n = 4) were excluded in the final analysis. The enrolment extended from March 2013 to July 2014. Respondents were either of the parents or a caregiver who is a close relative.

Written informed consent was obtained from the parents of the children and was duly signed by a witness. The consent contained the title, purpose, methods employed in the study, benefits to the child as well as family and the interest of the respondent to participate on a voluntary basis to the study. The confidentiality of the study during the analysis was also mentioned in the consent. The consent process and study protocol was approved by the Institutional Ethics Committee (Title: The Chairperson, Institutional Ethics Committee, Amrita Institute of Medical and Research Centre, AIMS—Ponekkara Post, Kochi, Kerala– 682 041. Registration Number: ECR/129/Inst/KL/2013. Details of registration available at http://www.cdsco.nic.in/Forms/list.aspx?lid=1911).

### Data Collection

A pilot tested semi structured interview schedule was used to collect information from the respondents. Demographic and patient birth details were collected from the parents / caregivers. Details of cardiac ailments, surgical procedure and direct hospital costs were collected from the hospital database. Direct costs included pharmacy, materials, services (surgery, consultation etc), diagnostic tests, bed charges, nursing charges and other miscellaneous charges. In addition, indirect cost that included travel, stay, food expenses and salary loss of bystanders were collected through direct interview. The interview was conducted by study personnel in the language preferred by the parent / caregiver (Malayalam, English/Tamil). Risk Adjustment in Congenital Heart Surgery –1 (RACHS–1) was used to classify the complexity of congenital heart disease surgery [8]. Socio economic class was determined using modified Kuppuswamy socio economic scale [9]. Information on the means of meeting the incurred expenses, various support systems availed and the level of perceived financial stress was also collected.

Follow up data at 6 months after surgery were collected from 557 patients. Follow up visits were pending for 67 subjects at this time (total eligible for follow up, n = 624, follow up coverage 89.3%). A modified version of the questionnaire used at baseline was re-administered during the follow up visit.

### Statistical Analysis

We summarized demographic and social-economic variables to characterize the study population (Table 1). For normally distributed continuous variables we presented the mean and standard deviation. For categorical variables we presented the proportion. In situations where the continuous variables were skewed we reported the median and interquartile range. The relationship between RACHS, hospital stay days (LOS), ICU stay days, and social-economic status with family cost of congenital heart surgery was evaluated using the ordinary least square (OLS) linear regression model, adjusting for potential confounding variables. Three types of costs were analysed as response variables: the total cost, the direct cost, and the indirect cost. Since the costing variables were highly skewed, they were log-transformed to meet the normality assumptions required by the OLS linear regression model. The resulting parameter estimates were then back transformed to their original scale to facilitate the interpretation of the results. The back-transformed parameters can be interpreted as “median ratio” [10]. The regression residual errors were examined for the constancy of variance using the graphs of residuals plotted against the predicted values. The cut-off point for statistical significance was set at an α-level of 5%. The 95% confidence intervals were also reported. The statistical analysis was done using SPSS version 17.

**Table 1:** Patient Characteristics.

	n (%)	Median (IQR)
Age (months)		8.2 (3.0–36.0)
Hospital stay (days)		13.0 (10.0–18.0)
ICU stay (days)*		4.0 (2.0–7.0)
Ventilation duration (days)^#^		1.0 (0.7–2.5)
**Gender**—Male	371 (57.6)	
**Place of residence—**Rural	491 (76.2)	
**Order of birth**		
I	337 (52.3)	
II	232 (36.0)	
III	63 (9.8)	
IV	10 (1.6)	
V	2 (0.3)	
**Socio-economic Class**		
Lower	15 (2.3)	
Upper lower	100 (15.5)	
Lower middle	230 (35.7)	
Upper middle	277 (43.0)	
Upper	22 (3.4)	
**RACHS** ^**$**^ **Category**		
I	104 (16.1)	
II	306 (47.5)	
III	163 (25.3)	
IV	68 (10.6)	
V	0	
VI	3 (0.5)	

* Details of ICU stay were not available for 24 subjects at baseline.

^#^ Details of Ventilation time were not available for 21 subjects at baseline.

^$^ Risk Adjustment for Congenital Heart Surgery

## Results

### Patient characteristics

The characteristics of study patients are presented below in Table 1. Among the 670 patients who met inclusion criteria, 22 families (3.3%) did not consent to participate and 4 patients (0.6%) died during the immediate postoperative hospital stay. The data of 644 consecutive patients undergoing surgery for congenital heart defect were analysed for the study. There were 371 male (57.6%) patients. The median age of the patients was 8.2 months (IQR: 3.0–36.0 months). The largest proportion of families (43.0%) belonged to upper middle class. Costs are reported in Indian rupees (INR) and International dollars (I$). Conversion rates as of June 7 2014 have been used. The median annual family income of the patients was INR 229000 (IQR 144000–360000) [I$ 13599 (IQR: 8551–21378)].

### Treatment expenses

The median total hospital cost (direct and indirect costs) was INR 201898 (IQR: 163287–266139) [I$ 11989 (IQR: 9696–15804)]. The median direct hospital cost for the patients was INR 179167 (IQR: 146868–233584) [I$ 10639 (IQR: 8721–13871)]. The median indirect hospital expenses was INR 18850 (IQR: 11713–29099) [I$ 1119 (IQR: 696–1728)]. The total expenses for each surgery risk category are presented in Table 2. There was an increasing trend for total treatment expenses from lower to higher RACHS categories (p < 0.001). Sub-group comparisons showed significant inter-group differences in total hospital expenses (p < 0.001) except between RACHS 3 and 4 (p = 0.611). The details of indirect expenses based on RACHS Categories are presented as supplementary information table (S1 and S2 Tables). The total expenses for each socio-economic category are also presented in Table 2. There was an increasing trend for total treatment expenses from lower to higher SES categories (p = 0.008).

**Table 2:** Expenses related to congenital heart surgery with respect to surgical risk category and Socio economic class.

	Total Hospital Expenses*	
	INR	I$**
	Median (IQR)	Median (IQR)
**RACHS**		
1	142863 (123549–170059)	8484 (7337–10099)
2	189707 (166211–234820)	11265 (9870–13944)
3	266020 (211235–313350)	15797 (12544–18607)
4 & 6	269822 (211399–349974)	16023 (12553–20782)
**Socio economic class**		
Lower	185189 (156139–248303)	10997 (9272–14745)
Middle	203940 (164188–270331)	12111 (9750–16053)
Upper	212974 (165495–352916)	12647 (9828–20957)
**All**	201898 (163287–266139)	11989 (9696–15804)

* Without excluding the amount of financial support

**Based on 2010 rates

The majority of families found alternate sources of financing for meeting the expenses related to the surgery (Table 3). A total of 254 (39.4%) families received financial support from sources that included the hospital patient support scheme which provides subsidised treatment,(22.4%), support from societies, clubs, relatives and friends. Only 75 families (11.6%) were able to cover the total hospital costs themselves without any external support. Among the families, only 25 (3.9%) had some form of insurance to support the costs.

**Table 3:** Alternate sources of finances adopted by families to meet the expenses related to child’s surgery.

Sources	Number (%)
Borrowing money from friends or relatives	321 (49.8)
Pledging gold	219 (34.0)
Private loans	67 (10.4)
Selling gold	33 (5.1)
Pledging property	19 (3.0)
Other sources	39 (6.1)

### Hospital expenses in relation to annual family income

The ratio of total hospital expenses to annual family income (HEI ratio) of each patient was calculated. The median HEI ratio was 0.93 (IQR: 0.52–1.49). There were four main categories of patients when the sources of funding of hospital expenses were taken into account- a) those who could pay the expenses in full by self b) those who paid the total expenses by arranging funds through personal initiatives like loans, pledging or selling of gold, property etc. c) those who received external financial support that included hospital concessions, support from friends and relatives, organisations etc. without the need to pay back d) those who were covered under the insurance or re-imbursement schemes. The patient category who received an amount < 10% of the total expense as financial support were excluded from category c and were included in the appropriate category. Also, those who received partial external financial support were included in category c, even if they arranged some of their funds as mentioned in category b. The median HEI ratio of these four groups were 0.39 (0.22–0.61), 0.86 (0.55–1.39), 1.21 (0.82–1.80) and 0.49 (0.37–0.76) respectively.

### Economic situation of the families

Among the families studied, 500 families (77.6%) reported debts related to the child’s disease. Among them, 212 (42.4%) reported that the debts related to child’s disease were more than 75% of their total debts and 365 (73.0%) reported that they had specific time limits to repay the debts.

### Productivity losses

The family based productivity losses related to the child’s illness were analysed for the study. Among parents and caregivers, 5.1% of fathers, 2.2% of mothers and 1.2% of other relatives had to quit their jobs due to child’s condition. The median loss of job days was 15 (IQR: 11–24) and median loss of man days was 35 (IQR: 24–50) during the child’s hospital admission period for surgery. The median loss of salary during the period was INR 8000 (IQR: 4500–15000) [I$ 475 (IQR: 267–891)].

### Perceived financial stress of the families

Perceived financial stress of the parent during the admission period of the child was reported as overwhelming (20.3%), high (33.7%), moderate (16.0%), little (21.7%) and no stress (8.2%). Average HEI ratio of families with high levels (overwhelming and high combined) of perceived stress was significantly high when compared to those with moderate (1.17 v/s 0.79, p = <0.001) and low levels (little and no stress combined) of perceived stress (1.17 v/s 0.66, p<0.001).

### Prediction models for total treatment expenses

The details from the prediction models constructed for total, direct and indirect costs are presented in Tables 4, 5, and 6. Compared to RACHS IV and above category, the median total cost was found to be 49% lower for children in RACHS I category and 27% lower for children in RACHS II category. The median total cost increased by 3% for each additional day of hospital stay (LOS). The same increased by 4% for each additional day of stay in intensive care unit (ICU). Compared to lower socio economic group, the median total cost was 23% higher for upper socioeconomic group and 9% higher for middle socioeconomic group. The details are available in Table 4.

**Table 4:** Relationship between risk factors and family total cost of congenital heart surgery.

	Median ratio [95% CI]; p-value	
	Crude	Adjusted
RACHS category (n = 644)^1^		
I	0.54 [0.49–0.59];<0.001	0.51 [0.47–0.56];<0.001
II	0.73 [0.67–0.79];<0.001	0.73 [0.68–0.79];<0.001
III	0.95 [0.87–1.03]; 0.221	0.95 [0.87–1.03]; 0.202
IV and above	1.00	1.00
Hospital stay days (n = 644)^2^		
Each additional day	1.03 [1.03–1.03];<0.001	1.03 [1.02–1.03];<0.001
ICU stay days (n = 620)^3^		
Each additional day	1.05 [1.04–1.05];<0.001	1.04 [1.03–1.04];<0.001
Socio-economic status (n = 644)^4^		
Upper	1.26 [1.07–1.49]; 0.006	1.23 [1.04–1.46]; 0.014
Middle	1.09 [1.01–1.18]; 0.019	1.09 [1.01–1.17]; 0.027
Lower	1.00	1.00

^1^Adjusted for age and annual income;

^2^adjusted for RACHS, age, and annual income;

^3^adjusted for RACHS, age, and income;

^4^adjusted for residential status.

**Table 5:** Relationship between risk factors and family direct cost of congenital heart surgery.

	Median ratio [95% CI]; p-value	
	Crude	Adjusted
RACHS category (n = 644)^1^		
I	0.53 [0.48–0.59];<0.001	0.51 [0.46–0.55];<0.001
II	0.73 [0.67–0.78];<0.001	0.73 [0.68–0.79];<0.001
III	0.95 [0.88–1.03]; 0.220	0.95 [0.87–1.03]; 0.102
IV and above	1.00	1.00
Hospital stay days (n = 644)^2^		
Each additional day	1.03 [1.03–1.03];<0.001	1.03 [1.02–1.03];<0.001
ICU stay days (n = 620)^3^		
Each additional day	1.05 [1.04–1.05];<0.001	1.04 [1.03–1.04];<0.001
Socio-economic status (n = 644)^4^		
Upper	1.17 [0.99–1.37]; 0.065	1.15 [0.97–1.35]; 0.101
Middle	1.05 [0.98–1.13]; 0.189	1.05 [0.97–1.12]; 0.226
Lower	1.00	1.00

^1^Adjusted for age and annual income;

^2^adjusted for RACHS, age, and annual income;

^3^adjusted for RACHS, age, and income;

^4^adjusted for residential status.

**Table 6:** Relationship between risk factors and family indirect cost of congenital heart surgery.

	Median ratio [95% CI]; p-value	
	Crude	Adjusted
RACHS category (n = 644)^1^		
I	0.53 [0.43–0.66];<0.001	0.53 [0.43–0.66];<0.001
II	0.71 [0.59–0.86];<0.001	0.77 [0.64–0.91]; 0.003
III	0.92 [0.75–1.12]; 0.409	1.02 [0.84–1.24]; 0.823
IV and above	1.00	1.00
Hospital stay days (n = 644)^2^		
Each additional day	1.03 [1.03–1.04];<0.001	1.03 [1.02–1.04];<0.001
ICU stay days (n = 620)^3^		
Each additional day	1.05 [1.04–1.06];<0.001	1.04 [1.02–1.05];<0.001
Socio-economic status (n = 644)^4^		
Upper	1.77 [1.26–2.48];<0.001	1.72 [1.23–2.41]; 0.002
Middle	1.38 [1.19–1.60];<0.001	1.37 [1.18–1.59];<0.001
Lower	1.00	1.00

^1^Adjusted for age and annual income;

^2^adjusted for RACHS, age, and annual income;

^3^adjusted for RACHS, age, and income;

^4^adjusted for residential status.

The median direct cost also was found to be 49% lower for RACHS I and 27% lower for RACHS II categories than for RACHS IV and above category. The median direct cost showed a 3% increase for each additional day of hospital stay and 4% increase for each additional day of stay in ICU. There were no significant differences for the median direct cost with regard to different socioeconomic categories. The details are available in Table 5.

The median indirect cost was found to be 47% lower for RACHS I category and 23% lower for RACHS II category than for RACHS IV and above category. There was a 3% increase in median indirect cost for each additional day of hospital stay and 4% increase for each additional day of stay in ICU. The median indirect cost was 72% higher for upper socioeconomic group and 37% higher for middle socioeconomic group when compared to lower socioeconomic group. The details are available in Table 6.

### Follow up at six months

Data of 557 patients were collected at 6 months after surgery for congenital heart disease. A comparison of baseline and follow up details are presented as supplementary information table (S3 Table). Job adjustments during the follow up period were seen among caregivers in an attempt to cope with the current situation. Among caregivers, 12.0% of fathers, 4.7% of mothers and 2.2% of other relatives took up a new job during the follow up period. In addition, 28.5% of fathers, 4.7% of mothers and 0.9% of other relatives started working additional days. Corresponding figures were 33.8%, 6.8% and 2.2% respectively for working longer hours. Among caregivers, 6.3% of fathers, 2.9% of mothers and 0.9% of other relatives reported working fewer days in the follow up period. In addition, 2.5% of fathers and 3.9% of mothers reported quitting their job in the follow up period.

Among the 557 families assessed during follow up, 290 (52.1%) reported that they borrowed money after the child’s hospital discharge. The reasons reported included-meeting treatment expenses or to take care of the child (57.2%) and repayment of earlier loan or debt (28.6%).

Financial support received after the child’s discharge was analysed as part of the study. Among the families, 62 (11.1%) received financial help from relatives or friends, 28 (5.0%) received support from people’s representative’s funds and 3 (0.6%) received support from other sources such as media and employer during the post-operative follow up period. In addition, 6 (1.1%) received funds from voluntary organizations during this period. Among families followed up at six months, 56.3% families reported that they have brought in some changes in their daily living like avoiding/ reducing expensive food items, entertainment activities and purchase of new clothes.

## Discussion

Developments in techniques for the diagnosis and management of CHD over the last fifty years have improved expected survival into adulthood to 85 percent [11]. Unfortunately, these developments have not been accompanied by reductions in costs of care. An infant born with heart disease requires about $60,000 in medical care to reach 21 years of age as estimated by a study from the United Kingdom [11]. The cost of care of patients in India, although lower, is still well beyond the reach of most Indian families [6].

This study attempts to provide data on the micro-economic impact from the perspective of individual families of children affected with CHD from a limited resource environment. The costs related to surgery for congenital heart disease are significant enough for the vast majority of families to forfeit a major proportion of their annual income to manage the situation. This is despite the fact that congenital heart disease surgery in India costs only a fraction of corresponding treatment expenses from developed nations [12]. Comparison of current study data with a recently published study from US suggests that surgical costs in India are less than 10% of those experienced in US for corresponding risk categories [12].

Out of the families studied, only one in 9 had enough savings to meet the surgery expenses and only one in 25 was covered by insurance schemes. Even though many were supported by various financial support schemes, more than a third of families had to resort to distress financing by selling or mortgaging valuable assets or seek loans to manage the expenses by themselves. Those who received partial support also had to manage the remaining costs through personal initiatives. One in three families resorted to pledging gold or property for money and one in 10 took private loans to arrange funds for surgery. These two options are known for higher interest rates and strict repay schedules in the Indian context. Failing the repay schedules are often associated with severe punitive measures (loss of pledged property/gold, interest on interest and non-repaying fines) adding to the financial stress of these families who took this route of funding.

Economic constraints could be one of the reasons for low representation of patients from lower socio economic strata reporting for surgical correction in the current study. This was despite the availability of supportive schemes to help them cover the medical expenses. In low and middle-income countries, where generally most health care costs are borne by patient families themselves, persistence or recurrence of impoverishment can happen for those facing poverty when they have to meet large, out-of-pocket expenses. These patterns have implications for national economic growth and poverty-reduction efforts [13].

Important differences exist between high-income and most low and middle-income nations with respect to health care delivery, particularly for high-end tertiary care. There is very little by way of health insurance for the overwhelming majority of Indian families. Most of the existing insurance schemes specifically indicate congenital defects as an exception in the list of conditions covered. Those who are covered often come in the purview of employer assured insurance, which are in very low proportions.

Families who have a child undergoing surgery for congenital heart disease are faced with the twin financial burden of direct hospital expenses as well as indirect expenses for food, transportation, accommodation and loss of salary of the caregivers. The latter part, which cannot be ignored, is often invisible to the medical treatment team. This study found that on an average two weeks are lost in the form of job days and over a month as man-days per child operated for cardiac illness. This productivity loss is significant considering the fact that that majority of these families are likely to have limited financial reserves.

There are several non-modifiable factors that are related to cost of congenital heart surgery. The two main ones include the complexity of the required surgical procedure and the preoperative comorbidity status of the child [5, 14]. The results suggest that the total expense increased with more complex surgeries. Connor et al also has reported that patients who had greater disease complexity were more likely to result in high resource utilization [5]. The costs of operation are also related to preoperative condition of the child. A number of additional factors that include neonatal sepsis, late presentation with pulmonary hypertension, advanced hypoxia are common in resource poor environments and contribute to duration of intensive care and hospitalization [14].

The prediction model for hospital costs related to congenital heart disease surgery suggests that surgical risk category is the major determinant for higher costs. Additional days of hospital stay and ICU stay also play a significant role in producing higher costs. Of the factors that predict higher costs, duration of hospital stay (LOS) and ICU stay are the only factors that are modifiable. A recent study from US states that 23% of the variation in costs across operating cardiac centers was attributed to by length of hospital stay [12]. This varied from as low as 2.6% for simple surgeries like ASD repair to as high as 45.5% for complex surgeries like Hemi Fontan [12]. These findings suggest that there is an opportunity to reduce cost of surgery by shorter hospital stay especially for complex surgeries.

The study findings underscores the need for attempts to reduce pre and post-operative hospital stay duration during surgical treatment for congenital heart disease as a cost saving intervention in low resource settings like India. Recommended approaches to contain the costs of surgical correction of congenital heart disease like timely referral, early surgical correction and specific strategies for postoperative care needs to be practiced to improve outcomes and to reduce treatment expenses [15]. Additionally, a number of surgical improvisations that have reported from programs with limited resources in the developing world need to be considered after adequate testing and validation [16]. Standardized fast track protocols of recovery care involving practices aimed at early extubation and mobilization in children undergoing certain types of heart surgery can also be utilized for the reduction of length of hospital stay and associated cost factors [17, 18].

The study also sheds light on two interesting factors. Our results suggest that the mean length of stay (LOS) is shorter by 6 days in our study when compared to a recently published large database of 15,453 congenital heart disease surgeries reported from US [19]. The results also suggest that the incremental cost from an additional 2 to 3 days of stay above the median LOS in a western context (12,718 surgeries, 27 centers, US) is more than enough to cover the total surgical cost of the same surgery from a low resource setting like the current study center [20].

Socio economic status appears to have a significant role in generating higher indirect costs. The prediction model suggests that a strong socio economic gradient is seen for indirect costs across various levels of SES. On the contrary, direct costs remain relatively stable across SES categories.

The current study points to the fact that many parents had to make adjustments in their job settings not only during the surgery, but also during the follow up period after surgery. Quitting jobs or working less could be for giving better attention to the child. Parents (especially fathers) resorting to working more days in the post-surgery period could probably be an effort to compensate for the financial loss. Economic woes appear to continue for most parents. This is evident from the fact that one out of two families have borrowed money in the follow up period to cope up with their child’s treatment demands. The gravity of the financial burden is such that one out of three families stated that more than three fourths of their cumulative debt is related to their child’s treatment. In addition, families are also incurring additional debts during follow up to pay out earlier debts with timelines attached to payment. This vicious cycle of debt leading to further debt is very distressing from a family perspective.

Financial situation is an important determinant of quality of life of parents with children having congenital heart disease [21]. The financial support extended from the community seems to be inadequate to majority of families who have a child undergoing surgery for heart disease.

The results of our study have several policy implications. We present direct and indirect cost details across a broad sample of congenital cardiac operations of various degrees of complexity. We have also presented the modifiable predictors of direct and indirect cost components of congenital cardiac surgery that can be explored as opportunities for cost reduction. Our results also document the productivity losses related to this illness. The results also stress the need for scaling up of support systems both from government as well as non-governmental agencies.

### Limitations

This is a single center study and the results may not adequately represent the population in the region. It is likely that the poorest did not get to reach the hospital at all. There is a distinct possibility that congenital heart disease may not be easily identified in many children from families belonging to the poorest sections of the society. Additionally, economic barriers may not allow families from poorest sections to reach tertiary institutions. Inaccuracies in reporting of family income and indirect expenses by the parents cannot be entirely ruled out. The usage of hospital expenses to annual family income (HEI ratio) is a new method adopted by us and may have its limitations due to inaccurate reporting of family income mentioned above.

## Conclusions

This study attempts to provide an estimate of expenses and utilization of available financial resources related to surgical treatment for congenital heart disease and provides data that could serve as a guide to public insurance schemes. The study points to the fact that there should be greater efforts to reduce the costs related to this condition. Wider community initiatives to support at least those families from the lower socioeconomic backgrounds are required to minimize the overall financial burden of congenital heart diseases.

## Supporting Information

S1 Appendix Tools for data collection- Economic Impact- Baseline. (DOC)

S2 Appendix Tools for data collection- Economic Impact- 6 month follow up. (DOC)

S1 Table Details of Indirect Expenses according to RACHS* Categories in INR^#^. (DOC)

S2 Table Details of Indirect Expenses according to RACHS* Categories in I$^#^. (DOC)

S3 Table Comparison of Patient Characteristics at Baseline and Follow up. (DOC)
