# Supplementary material for: Micro-Economic Impact of Congenital Heart Surgery: Results of a Prospective Study from a Limited-Resource Setting
Source: PLoS One. 2015 Jun 25;10(6):e0131348. doi: 10.1371/journal.pone.0131348 (PMC4482148; doi:10.1371/journal.pone.0131348)
Supplement: S1 Appendix — (DOC) [file pone.0131348.s001.doc]

**S1 APPENDIX**

**Tools for data collection.**

**ECONOMIC IMPACT - Baseline**

1. **How do you feel about your current financial situation?**

1) Very Comfortable 2) Comfortable 3) Moderately Comfortable

4) Somewhat Comfortable 5) Not Comfortable at All

1. **What is the level of your financial stress today?**

1) No stress 2) A little stress 3) A moderate amount of stress

4) A high amount of stress 5) An overwhelming amount of stress

3. **Please rate any difficulties you have experienced in daily living following your surgery?**

**3a.** **Obtaining Food**

1) No difficulty 2) Mild difficulty 3) Moderate difficulty 4) Extreme difficulty 5) Don’t Know

**3b. Obtaining Clothing**

1) No difficulty 2) Mild difficulty 3) Moderate difficulty 4) Extreme difficulty

5) Don’t Know

**3c. Obtaining Shelter**

1) No difficulty 2) Mild difficulty 3) Moderate difficulty 4) Extreme difficulty

5) Don’t Know

**3d. Doing Other Household Activities (Entertainment, Purchases, Health related expenses etc...)**

1) No difficulty 2) Mild difficulty 3) Moderate difficulty 4) Extreme difficulty

5) Don’t Know

**3e. Children’s Education**

1) No difficulty 2) Mild difficulty 3) Moderate difficulty 4) Extreme difficulty

5) Don’t Know

**4. Since your surgery, how often do you worry about being able to meet normal monthly living**

**expenses?**

1) Never 2) Rarely 3) Sometimes 4) Always

**5. Due to a child’s illness, which family member did the following (check all that apply)**

|  | Myself | Spouse | Sibling(s) | Other family members | None |
| --- | --- | --- | --- | --- | --- |
| Started a new job |  |  |  |  |  |
| Worked more hours |  |  |  |  |  |
| Worked more days |  |  |  |  |  |
| Worked less |  |  |  |  |  |
| Stopped working |  |  |  |  |  |

**6. Do you have any debt currently?**

1) Yes 2) No

**7. If Yes, What kind of debt you have?**

(1) Housing loan (2) Vehicle loan (3) Educational loan (4) Agricultural loan

(5) Loan from friends & relatives (6) Other (specify) ___________________

**8. Is your debt related to your child’s illness?**

1) Yes 2) No

**9. If Yes, quantify how much it is?**

1) Below 25% of the total debt 2) Between 25%-50% of the total debt

3) Between 50%- 75% of the total debt 4) Above 75% of the total debt

5) Not Applicable

**10. Is there any time limit for repay the amount?**

1) Yes 2) No 3) Not Applicable

**11. If Yes, Specify the duration? --------------**

**12. How confident are you that you will be able to clear your current debt in a defined period of**

**time?**

1) Not at all confident 2) Not very confident 3) Moderately confident

4) Very confident 5) Extremely confident

**13. How often do you compromise your financial plans due to the child’s illness?**

1) Not at all 2) Some times 3) Most often 4) Always 5) Don’t Know
