# Supplementary material for: Micro-Economic Impact of Congenital Heart Surgery: Results of a Prospective Study from a Limited-Resource Setting
Source: PLoS One. 2015 Jun 25;10(6):e0131348. doi: 10.1371/journal.pone.0131348 (PMC4482148; doi:10.1371/journal.pone.0131348)
Supplement: S2 Appendix — (DOC) [file pone.0131348.s002.doc]

**S2 Appendix**

**ECONOMIC IMPACT – 6 month Follow up**

**1. How do you feel about your current financial situation?**

1) Very Comfortable 2) Comfortable 3) Moderately Comfortable

4) Somewhat Comfortable 5) Not Comfortable at All

**2. What is the level of your financial stress today?**

1) No stress 2) A little stress 3) A moderate amount of stress

4) A high amount of stress 5) An overwhelming amount of stress

**3. Please rate any difficulties you have experienced in daily living following the surgery?**

**3a.** **Obtaining Food**

1) No difficulty 2) Mild difficulty 3) Moderate difficulty 4) Extreme difficulty 5) Don’t Know

**3b. Obtaining Clothing**

1) No difficulty 2) Mild difficulty 3) Moderate difficulty 4) Extreme difficulty

5) Don’t Know

**3c. Obtaining Shelter**

1) No difficulty 2) Mild difficulty 3) Moderate difficulty 4) Extreme difficulty

5) Don’t Know

**3d. Doing Other Household Activities (Entertainment, Purchases, Health related expenses etc...)**

1) No difficulty 2) Mild difficulty 3) Moderate difficulty 4) Extreme difficulty

5) Don’t Know

**3e. Children’s Education**

1) No difficulty 2) Mild difficulty 3) Moderate difficulty 4) Extreme difficulty

5) Don’t Know

**4. Currently, how often do you worry about being able to meet normal monthly living expenses?**

1) Never 2) Rarely 3) Sometimes 4) Always

**5. Due to a child’s illness, in the last 6 months which family member did the following (check all that apply)**

|  | Father | Mother | Sibling(s) | Other family members | None |
| --- | --- | --- | --- | --- | --- |
| Started a new job |  |  |  |  |  |
| Worked more hours |  |  |  |  |  |
| Worked more days |  |  |  |  |  |
| Worked less |  |  |  |  |  |
| Stopped working |  |  |  |  |  |

**6. Did you have debt related to child’s surgery/ admission?**

1) Yes 2) No

**7. If yes, were you able to repay your debt?**

1) Completely paid 2) Correctly repaying the interest/ installments

3) Has paid some part / paid incorrectly 4) Never paid any part of the debt

**8. Did you borrow money / take loan from any source after the child’s discharge?**

1) Yes 2) No (please move to Q. No: 11)

**9. If yes, from which source?**

1) Gold loan 2) Selling of gold 3) Property loan 4) Selling of property

5) Friends and relatives 6) Employer 7) Others…………….

**10. If yes, for what reason?**

1) To meet the daily living expenses 2) To repay the earlier loan/ debt

3) To meet the treatment expenses/ care of the child

4) Any other reason …………………………………

**11. Did you receive any financial support after the child’s surgery?**

1) No support 2) Relatives and friends 3) Voluntary organizations, clubs 4) Media 5) Prime Minister/ Chief Minister/ MP /MLA fund

6) Insurance/Reimbursement 7) Any other…………….

**12. If support received, how much? …………………….**

**13. Do you change your future financial plans after the child’s surgery?**

1) Not at all 2) Some times 3) Most often 4) Always 5) Don’t Know

**14. Do you feel that the money spent for the surgery was worth?**

1) Yes 2) No 3) Don’t know

**15. If no, please state the reasons? .........................................................................................**

**16. Did you make any change in the daily living after the child’s surgery?**

1) No change 2) Avoid/ reduce entertainment activities

3) Avoid expensive food items 4) Avoid/ reduce purchase of clothes

5) Any other.................................................

**17. Did you stay in the guest house or outside rented room after the discharge (when admitted for surgery) from the hospital?**

1) Yes 2) No

**18. If yes, how many days? ................**

**19. If yes, how much did it cost? ......................**

**ACTIVITY**

**1. Do you restrict the child’s activity currently?**

1) Yes 2) No

**2. After the child’s surgery, do you or any of your family members engage more in**

1) Religious activities 2) Sports/ Leisure activities

3) Spending time with family 4) Not applicable

**If the child is above 4 years, kindly answer the following questions:**

**3. Did your child have adequate information about her/his heart surgery before the surgery?**

1) Yes 2) No

**4. Do you feel any behavioral change in the child after the surgery?**

1) No change 2) More confident

3) Less confident 4) More social mingling

5) Less social mingling 6) Demands more care and attention

7) Easily irritated 8) Any other…………………………

**5. Do you feel that your child’s academic performance has changed after the surgery?**

1) No change 2) Better in studies

3) Decline in academic performance 4) Not applicable

**6. Do you feel any change in the activity pattern of the child after the surgery?**

1) No 2) More active 3) Less active
