# Supplementary material for: Micro-Economic Impact of Congenital Heart Surgery: Results of a Prospective Study from a Limited-Resource Setting
Source: PLoS One. 2015 Jun 25;10(6):e0131348. doi: 10.1371/journal.pone.0131348 (PMC4482148; doi:10.1371/journal.pone.0131348)
Supplement: S1 Table — (DOC) [file pone.0131348.s003.doc]

**Supplementary Table**

**S1 Table: Details of Indirect Expenses according to RACHS^*^ Categories in INR^#^**

| **RACHS** | **Stay**  **n**  **Median**  **(IQR)** | **Travel**  **n**  **Median**  **(IQR)** | **Food**  **n**  **Median**  **(IQR)** | **Loss of salary**  **n**  **Median**  **(IQR)** | **Miscellaneous**  **n**  **Median**  **(IQR)** | **Total**  **n**  **Median**  **(IQR)** |
| --- | --- | --- | --- | --- | --- | --- |
| **1** | 104 | 100 | 104 | 69 | 82 | 104 |
|  | 2300•0 | 2000•0 | 2550•0 | 6600•0 | 0•0 | 12275•0 |
|  | (1212•5- 3500•0) | (525•0- 4812•5) | (2000•0- 3750•0) | (4000•0- 20000•0) | (0•0- 500•0) | (8287•5- 23807•5) |
| **2** | 305 | 300 | 305 | 249 | 252 | 306 |
|  | 3150•0 | 2550•0 | 3600•0 | 7500•0 | 0•0 | 17420•0 |
|  | (1845•0- 5000•0) | (1400•0- 5000•0) | (2475•0- 5200•0) | (4300•0- 12000•0) | (0•0- 1000•0) | (10976•5- 26775•0) |
| **3** | 162 | 162 | 163 | 130 | 144 | 163 |
|  | 4450•0 | 4000•0 | 5000•0 | 10000•0 | 500•0 | 22850•0 |
|  | (2575•0- 7000•0) | (2000•0- 7000•0) | (3360•0- 7400•0) | (5150•0- 15000•0) | (0•0- 1000•0) | (15650•0- 33000•0) |
| **4** | 71 | 68 | 71 | 58 | 56 | 71 |
|  | 4550•0 | 6000•0 | 5250•0 | 8200•0 | 0•0 | 23800•0 |
|  | (2500•0- 6300•0) | (2000•0- 8000•0) | (3300•0- 7200•0) | (5750•0- 18125•0) | (0•0- 500•0) | (15850•0- 37150•0) |
| **Total** | 642 | 630 | 643 | 506 | 534 | 644 |
|  | 3500•0 | 3000•0 | 3840•0 | 8000•0 | 0•0 | 18850•00 |
|  | (1880•0- 5325•0) | (1350•0- 6000•0) | (2500•0- 5760•0) | (4500•0- 15000•0) | (0•0- 1000•0) | (11712•5- 29099•0) |

*RACHS – Risk Adjustment for Congenital Heart Surgery

# INR- Indian Rupees
