# Supplementary material for: Micro-Economic Impact of Congenital Heart Surgery: Results of a Prospective Study from a Limited-Resource Setting
Source: PLoS One. 2015 Jun 25;10(6):e0131348. doi: 10.1371/journal.pone.0131348 (PMC4482148; doi:10.1371/journal.pone.0131348)
Supplement: S2 Table — (DOC) [file pone.0131348.s004.doc]

**Supplementary Table**

**S2 Table: Details of Indirect Expenses according to RACHS^*^ Categories in I$^#^**

| **RACHS** | **Stay**  **n**  **Median**  **(IQR)** | **Travel**  **n**  **Median**  **(IQR)** | **Food**  **n**  **Median**  **(IQR)** | **Loss of salary**  **n**  **Median**  **(IQR)** | **Miscellaneous**  **n**  **Median**  **(IQR)** | **Total**  **n**  **Median**  **(IQR)** |
| --- | --- | --- | --- | --- | --- | --- |
| **1** | 104 | 100 | 104 | 69 | 82 | 104 |
|  | 137•0 | 118•8 | 151•4 | 391•9 | •0 | 728•9 |
|  | (72•0- 208•0) | (31•0- 286•0) | (119•0- 223•0) | (238•0- 1188•0) | (0•0- 30•0) | (492•0- 1414•0) |
| **2** | 305 | 300 | 305 | 249 | 252 | 306 |
|  | 187•0 | 151•0 | 214•0 | 445•0 | 0•0 | 1034•0 |
|  | (110•0- 297•0) | (83•0- 297•0) | (147•0- 309•0) | (255•0- 713•0) | (0•0 - 59•0) | (652•0- 1590•0) |
| **3** | 162 | 162 | 163 | 130 | 144 | 163 |
|  | 264•0 | 238•0 | 297•0 | 594•9 | 30•0 | 1357•0 |
|  | (153•0- 416•0) | (119•0- 416•0) | (200•0- 439•0) | (306•0- 891•0) | (0•0- 59•0) | (929•0- 1960•0) |
| **4** | 71 | 68 | 71 | 58 | 56 | 71 |
|  | 270•0 | 356•0 | 312•0 | 487•0 | 0•0 | 1413•0 |
|  | (149•0- 374•0) | (119•0- 475•0) | (196•0- 428•0) | (341•0 - 1076•0) | (0•0- 30•0) | (941•0- 2206•0) |
| **Total** | 642 | 630 | 643 | 506 | 534 | 644 |
|  | 208•0 | 178•0 | 228•0 | 475•0 | 0•0 | 1119•0 |
|  | (112•0- 316•0) | (80•0- 356•0) | (149•0- 342•0) | (267•0- 891•0) | (0•0- 59•0) | (696•0- 1728•0) |

*RACHS- Risk Adjustment for Congenital Heart Surgery

# I$- International Dollars as of 2010
