# Supplementary material for: Micro-Economic Impact of Congenital Heart Surgery: Results of a Prospective Study from a Limited-Resource Setting
Source: PLoS One. 2015 Jun 25;10(6):e0131348. doi: 10.1371/journal.pone.0131348 (PMC4482148; doi:10.1371/journal.pone.0131348)
Supplement: S3 Table — (DOC) [file pone.0131348.s005.doc]

**Supplementary Table**

**S3 Table: Comparison of Patient Characteristics at Baseline and Follow up**

|  | **Baseline (n=644)** | **Follow up (n=557)** |
| --- | --- | --- |
| Age (months) | 8·2 (3·0 – 36·0) | 8·4 (3·0 – 36·0) |
| Hospital stay (days) | 13·0 (10·0 – 18·0) | 13·0 (10·0 - 18·0) |
| ICU stay (days) | 4·0 (2·0 – 7·0) | 4·0 (2·0 - 7·0) * |
| Ventilation duration (days) | 1·0 (0·7 – 2·5) | 1·0 (0·7 - 2·5) # |
| **Gender** - Male | 371 (57·6) | 322 (57·8) |
| **Place of residence -** Rural | 491 (76·2) | 431 (77·4) |
| **Order of birth** |  |  |
| I | 337 (52·3) | 290 (52·1) |
| II | 232 (36·0) | 205 (36·8) |
| III | 63 (9·8) | 51 (9·2) |
| IV | 10 (1·6) | 9 (1·6) |
| V | 2 (0·3) | 2 (0·4) |
| **Socio-economic Class** |  |  |
| Lower | 15 (2·3) | 14 (2·5) |
| Upper lower | 100 (15·5) | 89 (16·0) |
| Lower middle | 230 (35·7) | 197 (35·4) |
| Upper middle | 277 (43·0) | 238 (42·7) |
| Upper | 22 (3·4) | 19 (3·4) |
| **RACHS^$^ Category** |  |  |
| I | 104 (16·1) | 83 (14·9) |
| II | 306 (47·5) | 265 (47·6) |
| III | 163 (25·3) | 148 (26·6) |
| IV | 68 (10·6) | 59 (10·6) |
| V | 0 | 0 |
| VI | 3 (0·5) | 2 (0·4) |

* Details of ICU stay were not available for 23 subjects at follow up.

# Details of Ventilation time were not available for 20 subjects at follow up.

$ Risk Adjustment for Congenital Heart Surgery
